# Transition from physical activity to inactivity increases skeletal muscle miR‐148b content and triggers insulin resistance

**DOI:** 10.14814/phy2.12902

**Published:** 2016-09-05

**Authors:** Caroline Gastebois, Stéphanie Chanon, Sophie Rome, Christine Durand, Elise Pelascini, Audrey Jalabert, Vanessa Euthine, Vincent Pialoux, Stéphane Blanc, Chantal Simon, Etienne Lefai

**Affiliations:** ^1^ CarMeN Laboratory INSERM U1060 INRA 1397 University of Lyon 1 Oullins France; ^2^ Department of Digestive and Bariatric Surgery Hospices Civils de Lyon Lyon France; ^3^ Laboratoire LIBM EA 7424 University of Lyon Lyon France; ^4^ Institut Pluridisciplinaire Hubert Curien CNRS UMR 7178 University of Strasbourg Strasbourg France

**Keywords:** Insulin‐signaling pathway, miRNA, muscle tissue, myotube, physical inactivity

## Abstract

This study investigated miR‐148b as a potential physiological actor of physical inactivity‐induced effects in skeletal muscle. By using animal and human protocols, we demonstrated that the early phase of transition toward inactivity was associated with an increase in muscle miR‐148b content, which triggered the downregulation of NRAS and ROCK1 target genes. Using human myotubes, we demonstrated that overexpression of miR‐148b decreased NRAS and ROCK1 protein levels, and PKB phosphorylation and glucose uptake in response to insulin. Increase in muscle miR‐148b content might thus participate in the decrease in insulin sensitivity at the whole body level during the transition toward physical inactivity.

## Introduction

Current lifestyle changes, such as the increase in sedentary behaviors, are associated with higher prevalence of chronic metabolic diseases, whereas regular physical activity improves metabolic functions. Sedentary behaviors have been related to adverse health outcomes independently from physical activity (Tremblay et al. 2010; Grøntved and Hu 2011; Thorp et al. 2011; Edwardson et al. 2012; de Rezende et al. 2014). Examining the consequence of a reduction in physical activity at the whole body level metabolism, it was shown that physical inactivity, which is characterized by fewer skeletal muscle contractions, is associated with decreased fat oxidation capacity (Laye et al. 2009; Bergouignan et al. 2013), and impaired insulin sensitivity and glycemic control (Reynolds et al. 2015) in skeletal muscle. However, the molecular actors involved in the metabolic responses induced by physical inactivity are not completely understood; more particularly the role of novel regulators, such as the microRNAs (miRNAs) in the muscle tissue response has to be specified.

MiRNAs are small noncoding RNAs of about 18–24 bases long that negatively regulate gene expression, mainly at the posttranscriptional level. Functional analysis of miRNA target genes have shown that they play a major role in the regulation of developmental processes including cell growth and differentiation and programmed cell death (Ebert and Sharp 2012; Mendell and Olson 2012; Ivey and Srivastava 2015). Recently, it was demonstrated that miRNAs were also involved in the regulations of biological processes induced by physical activity. Both, acute and chronic exercise protocols modified the levels of miRNAs in blood, in skeletal muscle, in heart, or in central nervous system (Davidsen et al. 2011; Liu et al. 2012; Baggish et al. 2014). However, the effects might differ according to exercise intensity, duration, or type (i.e., resistance vs. aerobic) (Davidsen et al. 2011; Baggish et al. 2014). To date, only few studies have examined the role of miRNA levels upon transition toward physical inactivity and related biological process. Skeletal muscle miRNAs were explored after acute and short‐term exercise and endurance training (Russell et al. 2013), two bed rest studies involving human volunteers (Ringholm et al. 2011b; Rezen et al. 2014), and two studies on rodent adaptations during extreme inactivity or simulated weightlessness (Allen et al. 2009; McCarthy et al. 2009) induced changes in skeletal muscle miRNA content.

Recently, it was shown that inflammatory muscle catabolism leading to skeletal muscle wasting was associated with the increase in miR‐148b (Zhang et al. 2015). Interestingly, it was described that two of its targets, NRAS and ROCK1, were involved in insulin‐signaling and glucose metabolism. ROCK1 deficiency was found to induce insulin resistance by impairing insulin signaling in skeletal muscle in mice (Lee et al. 2009). ROCK1 was found to be a positive regulator of insulin action on glucose transport in adipocytes and muscle cells (Chun et al. 2012). Thus, in this study, we hypothesized that miR‐148b might be involved in metabolic responses induced by changes in the level of activity, in skeletal muscle.

## Materials and Methods

### Subjects and study protocol

**Figure 1:**
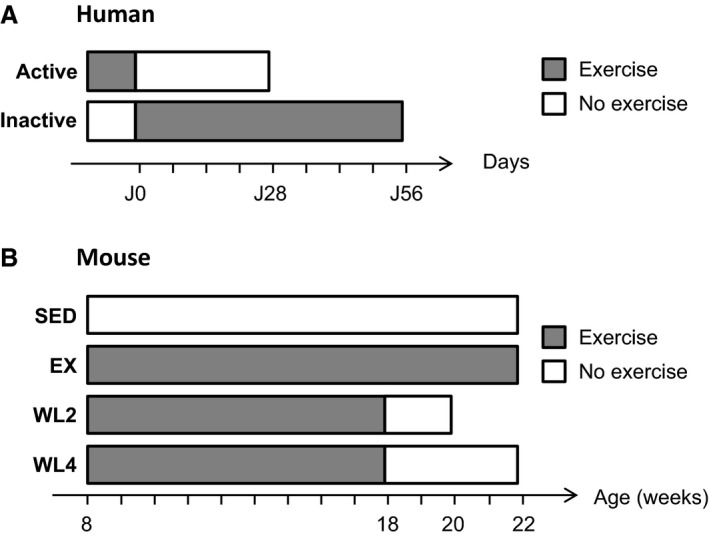
Timeline experiments. Grey blocks represent periods during which men were active (A) and mice had access to wheels (B); white blocks represent periods during which men were inactive (A) and wheels have been locked (B). (A) Active men were detrained during 4 weeks, whereas inactive men followed an 8‐week training. (B) Compared to nonexercised mice (SED), exercised mice (EX) were running throughout the experimental protocol. Detrained mice stopped running at 18 weeks of age either for 2 weeks (WL2) or for 4 weeks (WL4).

The protocol and main outcomes of the interventional protocol LIPOX are detailed elsewhere (Bergouignan et al. 2013). Briefly, 24 healthy men were included in the study and divided into two groups (*n *=* *12) according to their active or inactive status. Inactive men were submitted to 2 months of moderate physical training at current recommendations, that is, three 60‐min supervised sessions per week on a cycle ergometer and one additional session in free‐living conditions during weekends. Active men were submitted to 1 month of detraining, that is, suppression of all structured physical activities and reduction in spontaneous activities of daily living (Fig. 1A). Written consent was obtained from each subject prior involvement in this study. The study was approved by the Institutional Review Board of Alsace‐1. Characteristics of participants are summarized in Table 1. Pre‐ and postintervention *vastus lateralis* muscle biopsies were available for 12 participants (six active and six inactive).

**Table 1:** Characteristics of the participants before and after interventions on physical activity

Intervention	Active men		Inactive men	
	Detraining		Training	
	Before (*n *=* *6)	After (*n *=* *6)	Before (*n *=* *6)	After (*n *=* *6)
Anthropometric measures				
Age (years)	24.1 ± 1.2		25.5 ± 14	
BW (kg)	70.2 ± 3.1	69.5 ± 3.7	77.8 ± 2.1	77.6 ± 2.1
BMI (kg/m²)	22.1 ± 0.5	22.1 ± 0.7	23.6 ± 0.6	23.5 ± 0.6
FFM (kg)	59.6 ± 2.6	58.1 ± 3.2*	62.2 ± 1.5	62.2 ± 1.4
Physical activity outcomes				
VO_2 peak_ (mL/min/kg)	48.7 ± 2.1	45.1 ± 1.6*	39.9 ± 1.4†	43.9 ± 1.8*
AEE (Kj/d/kg)	87.6 ± 4.1	76.0 ± 3.8*	34.0 ± 3.5†	56.7 ± 4.2*

AEE, activity energy expenditure; BMI, body mass index; BW, body weight; VO_2peak_, peak oxygen uptake.

All values are means ± SEMs.

*P < 0.05 compared with baseline, ^†^ *P* < 0.01 compared with active men at baseline.

### Animals and experimental design

Twenty‐seven male C57BL/6 mice, 5 weeks old, were housed at standard conditions (22°C, 12/12 h dark–light cycle, food and water ad libitum). Mice were housed in individual cage equipped with a 15 cm radius voluntary running wheel outfitted with a BC800 bicycle computer (Sigma GmbH, Neustadt, Germany). Wheel rotation numbers were recorded daily to calculate the running distance (km/day). All experimental procedures were accepted by local ethic committee and performed in accordance to National and European legislation.

After 1 week of acclimatization, mice were randomly affected to one of the four experimental groups (Fig. 1B): no exercise (SED, *n *=* *6), exercise (EX, *n *=* *7), exercise followed by 2 weeks (WL2, *n *=* *7), or 4 weeks (WL4, *n *=* *7) of suppression of voluntary exercise obtained by locking wheels at 18 weeks of age. In all groups, body weight (g) and food intake (g/day) were measured twice a week.

### Culture of skeletal muscle cells

For human primary myotubes, *rectus abdominis* or *gluteus maximus* muscle biopsies were taken from metabolically healthy subjects during planned surgery (*n *=* *6, M/F = 3/3, age = 58.5 ± 4.4, BMI = 24.5 ± 2.0). All patients gave their written consent after being informed of the nature, purpose, and possible risks of the study. The experimental protocol (DIOMEDE) was approved by the Ethical Committees SUD EST IV. The myoblasts were purified and differentiated into myotubes according to the procedure previously described (Perrin et al. 2015).

### Modulation of miR‐148b expression in human primary muscle cells

Inhibition of miR‐148b expression was performed by using miRNA inhibitor (hsa‐miR‐148b‐3p miRCURY LNA microRNA inhibitor, 5`‐fluorescein labeled) and compared to control (Negative control A miRCURY LNA^TM^ microRNA inhibitor control, 5 nmol, 5′‐fluorescein labeled) from Exiqon (Vedbæk, Denmark). Fully differentiated myotubes (7 days) were transfected for 48 h with 100 pmols of miRNA inhibitor by using the Hiperfect transfection reagent (Qiagen, Courtaboeuf, France) according to the manufacturer's protocol.

Overexpression of miR‐148b was performed by using a plasmid expressing a pre‐miR‐148 (HmiR0185‐MR04, precursor miRNA expression clone for hsa‐mir‐148b) and compared to control (CmiR0001‐MR04, miRNA scrambled control clone for pEZX‐MR04) from GeneCopoeia (Labomics, Nivelles, Belgium). Fully differentiated myotubes (7 days) were transfected for 48 h with 2 *μ*g microRNA expression plasmid using the Exgen500 transfection reagent (Euromedex, Souffelweyersheim, France) according to the manufacturer's protocol.

For insulin‐signaling assays, myotubes were serum starved for one night before insulin stimulation (10 or 100 nmol/L) for 10 min.

### RNA isolation and quantification of miRNAs

RNA from −80°C frozen *gastrocnemius lateralis* mouse muscle or human primary myotubes was isolated by using the Tri Reagent (Sigma, St Quentin Fallavier, France). RNA from the −80°C frozen *vastus lateralis* human muscle biopsies was extracted using the mirVana miRNA Isolation Kit protocol (Life Technologies, Saint Aubin, France). To quantify miR‐148b, 50 ng total RNA was reverse transcribed in 15 *μ*L reactions using the TaqMan MicroRNA Reverse Transcription Kit (4366596, Life technology, Saint Aubin, France). cDNA was diluted 16× and assayed in 20 *μ*L PCR reactions using TaqMan Universal PCR Master Mix (4324018, Life technology, Saint Aubin, France) and a Rotor‐GeneTM 6000 (Qiagen, Courtaboeuf, France). miRNA probe used was hsa‐mir148b‐3p (000471, MI0000811) from Life technology (Saint Aubin, France). SLC2A4 (Glut4) and TBP (Tata Box Binding Protein) mRNA were quantified by RT‐qPCR using already published procedures (Bergouignan et al. 2013) with the respective primers: SLC2A4 sense 5′‐ GGGTTTCCAGTATGTTGCGG ‐3′; SLC2A4 antisense 5′‐ CTGGGTTTCACCTCCTGCTC ‐3′; TBP sense 5′‐TGGTGTGCACAGGAGCCAAG‐3′; and TBP antisense 5′‐ TTCACATCACAGCTCCCCAC‐3′.

### Protein expression analyses by Western blotting

Proteins were extracted and immunoblotted as previously described (Dessalle et al. 2012). All membranes were blocked with 4% BSA (Bovine Serum Albumin, Euromedex, Souffelweyersheim, France) and probed with anti‐N‐RAS (sc‐31, Santa Cruz Biotechnology, Palo Alto, CA), anti‐ROCK1 (sc‐5560, Santa Cruz), anti‐*α*‐Tubulin (T5168, Sigma, Lyon, France), anti‐phospho‐Ser^473^ Akt (#4060, Cell Signaling Technology, Leiden, the Netherlands), anti‐Akt (#4691, Cell Signaling), anti‐phospho‐Thr^202^/Tyr^204^ p44/42 MAPK (Erk1/2) (Cell Signaling), and anti‐p44/42 MAPK (Erk1/2) (Cell Signaling). The membranes were incubated with secondary corresponding antimouse (172‐1011, Bio‐Rad, Marnes‐la‐Coquette, France) or antirabbit (172‐1019, Bio‐Rad) HRP conjugated antibody. Signals were revealed with Immunodetection kit ECL Luminata Classico (Millipore) and the imager Molecular Image^®^ ChemiDoc^™^XRS+ (Bio‐Rad). Proteins quantification was achieved using ImageLab 3.0 (Bio‐Rad).

### Glucose uptake measurement

Human myotubes were serum starved for 3 h then incubated in X‐DPBS (MgCl_2_: 0.5 mmol/L, CaCl_2_: 0.9 mol/L, BSA: 0.2%) with 20 *μ*mol/L of 2‐deoxy‐[^3^H]‐D‐glucose (2 *μ*ci/mL) and 1 mmol/L glucose for 15 min. Incubations were performed with or without insulin stimulation (1 h, 100 nmol/L) and in the presence or absence of cytochalasin B (20 *μ*mol/L). After incubation, the medium was removed and cells were rapidly washed with cold X‐DPBS, prior to lysis in 0.05 mol/L NaOH. Cell content radioactivity was determined by liquid scintillation counting (Perkin Elmer 2900 TR, Courtaboeuf, France) and protein content was quantified by using the Bradford protein assay.

### Statistical analysis

Data are presented as means ± SEM for more than three independent experiments. The number is indicated in each Figure legend. For pair‐wise comparisons, Student's *t*‐test was used. For multiple comparisons, analysis of variance (ANOVA) statistics were applied. Differences were considered statistically significant when *P* < 0.05.

## Results

To explore miRNAs changes during the early phase of transition from an active status toward a less active behavior, we performed two complementary protocols: the LIPOX protocol in healthy lean human subjects submitted to transition from active to inactive status, and opposite (Fig. 1A); the SEDENT protocol in rodent with the wheel‐lock model using adult mice (Fig. 1B). Main biological characteristics after and before interventions are presented in Table 1 for humans, and in Table 2 for mice.

**Table 2:** Characteristics of mice at 18 weeks, prior interventions on physical activity, and at sacrifice.*

Variables	SED (*n *=* *6)	EX (*n *=* *7)	WL2 (*n *=* *7)	WL4 (*n *=* *7)
During training period (mean from weeks 10 to 18)				
BW (g)	26.01 ± 0.58	26.27 ± 0.61	26.16 ± 0.84	26.26 ± 0.53
Running distance (km/day)	–	6.34 ± 0.41	5.51 ± 0.50	6.47 ± 1.03
Food intake (g/day)	3.5 ± 0.1†	4.1 ± 0.1	3.9 ± 0.1	4.1 ± 0.1
At week 18				
BW (g)	27.0 ± 0.7	26.9 ± 0.8	27.7 ± 0.9	27.2 ± 0.7
Running distance (km/day)	–	3.67 ± 0.38	3,89 ± 0.22	4.35 ± 0.84
Food intake (g/day) from weeks 10 to 18	3.5 ± 0.1†	4.1 ± 0.1	3.9 ± 0.1	4.1 ± 0.1
At sacrifice				
BW (g)	28.7 ± 0.6	29.0 ± 0.5	28.5 ± 1.1	30.2 ± 0.9
Food intake (g/day)	3.2 ± 0.1	3.8 ± 0.1	3.2 ± 0.1	3.3 ± 0.1
Running distance (km/day)	–	3.21 ± 0.31	–	–
*Gastrocnemius lateralis* (mg)	147.7 ± 2.4	153.0 ± 3.5	151.3 ± 2.6	152.2 ± 3.3

BW, body weight; EX, exercise; WL2, exercise followed by 2 weeks detraining; WL4, exercise followed by 4 weeks detraining, SED, no exercise.

All values are means ± SEMs.

*Running distance and food intake were recorded per cage (one mouse per cage).

^†^ *P* < 0.05 compared with running mice (EX, WL2, and WL4). Weekly recorded data are presented in Figures S1 to S3.

In humans, inactive participants exhibited lower activity energy expenditure AEE and maximal oxygen consumption (VO_2peak_) than the active participants (Table 1). Training in inactive men improved AEE and VO_2peak_, whereas detraining in active men induced opposite changes. Transition from active to inactive status led to a moderate decrease in fat‐free mass (FFM), whereas no difference was induced with training (Table 1).

In the mice protocol, at week 18, all exercise mice had similar body weight, running distance, and food intake, whereas sedentary mice (SED) had a lower food intake compared to the running mice (Table 2). For the active mice, food intake was directly correlated with running activity, and after wheel lock, WL2 and WL4 mice spontaneously decreased their food intake which returned to the level of the SED mice (Figs. S1–S3). At sacrifice, there was no difference for Gastrocnemius muscle weight between groups. After 2 or 4 weeks of wheel lock, WL2 and WL4 mice did not present a significant increase in body weight, compared to SED or exercise mice (EX) (Table 2).

### Muscle miR‐148b content decreased in the early phase of transition toward inactivity

**Figure 2:**
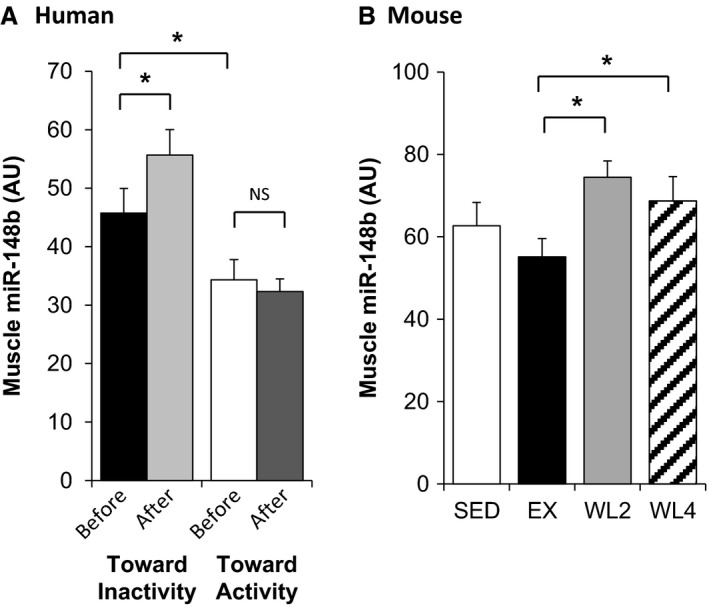
Increase in miR‐148b muscle content before and after activity status changes. (A) In active (*n* = 6) and inactive (*n* = 6) men before and after, respectively, 1 month of detraining or 2 months of training at current recommendations. (B) Muscle miR‐148b content in SED (*n* = 6), EX (*n* = 7), WL2 (*n* = 7), and WL4 (*n* = 7) mice assessed by qRT‐PCR. Values are means ± SEM. *indicates *P *<* *0.05, NS nonsignificant.

As major changes during physical activity or inactivity transition occur within the skeletal muscle tissue, we quantified miR‐148b changes in muscle samples during the early phase of transition toward inactivity. First, we used LIPOX study to quantify miR‐148b in muscle of active and inactive subjects before and after interventions. Results (Fig. 2A) showed that the 4 weeks detraining protocol induced an increase in miR‐148b muscle content in active subjects (+37%, *P* < 0.05). Despite significant lower expression contents before intervention in the inactive group, training did not significantly modify miR‐148b muscle content (Fig. 2A). In parallel, we quantified miR133a in the same muscle samples and did not find any significant differences, neither between the groups in the basal state nor induced by the protocols (Fig. S4).

We next quantified miR‐148b muscle content in mice submitted to a wheel‐lock protocol. Exercise mice (EX) were compared to mice for which wheels have been locked for 2 weeks (WL2), 4 weeks (WL4), or throughout the protocol (SED). As shown in Figure 2B, miR‐148b muscle content was increased in the WL2 group (+35%, *P* < 0.05) and the WL4 group (+25%, *P* < 0.05) compared to the group that continue to exercise (EX).

Taken together, these results showed that transition from active to inactive status triggered an increase in skeletal muscle content of miR‐148b both in human and animal models. Meanwhile, the reverse protocol (i.e., from inactive to active status) in humans did not induce changes in skeletal muscle miR‐148b content.

### ROCK1 and NRAS are muscle miR‐148b target genes

Among the target genes of miR‐148b predicted by mirPath (Vlachos et al. 2012), NRAS and ROCK1 have been previously validated in mammary tumor cell lines (Cimino et al. 2013). As these two proteins are involved in insulin‐signaling and glucose metabolism, we aimed at determining whether miR‐148b also regulated these two proteins in muscle.

**Figure 3:**
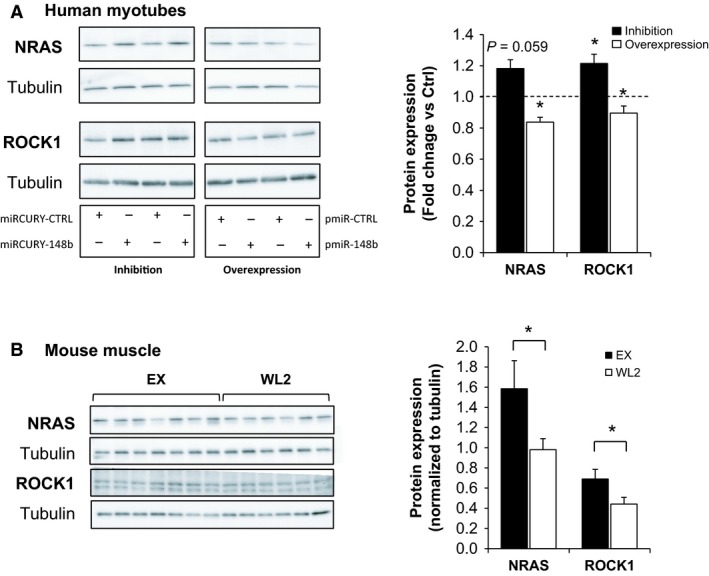
NRAS and ROCK1 are miR‐148b target genes. (A) Human primary differentiated myotubes were transfected with anti‐miR‐148b (miRCURY‐148b) or anti‐miR control (miRCURY‐CTRL), and with a plasmid expressing pre‐miR‐148b (pmiR‐148b) or with a control plasmid (pmiR‐CTRL). Left are presented illustrative Western blots; and right‐fold changes quantifications using *α*‐tubulin for normalization. (B) Mouse *Gastrocnemius* content in NRAS and ROCK1 proteins in EX (*n* = 7) and WL2 (*n* = 6) mice assessed by Western blotting. Left are presented illustrative Western blots, and right‐fold changes quantifications using g *α*‐tubulin for normalization. Values are means ± SEM. Results are presented as fold changes versus control. *indicates *P* < 0.05.

We thus modulated miR‐148b expression in in vitro experiments using muscle cells. Human primary differentiated myotubes were transfected with plasmid expressing a pre‐ or an anti‐miR‐148b. As shown in Figure 3A, both NRAS and ROCK1 proteins levels were increased when miR‐148b was inhibited (+18%, *P *=* *0.059 and +21%, *P *=* *0.049, respectively). Conversely, overexpression of miR‐148b decreased both NRAS and ROCK1 protein expression (−16%, *P *=* *0.035 and −11%, *P *=* *0.05 respectively).

We then quantified NRAS and ROCK1 proteins in mouse skeletal muscle, in the EX and WL2 groups, where the highest differences in miR‐148b were highlighted. As shown in Figure 3B, 2 weeks of forced inactivity triggered a decrease in both NRAS and ROCK1 proteins, concomitantly with the increase in miR‐148b.

These data validated that NRAS and ROCK1 were target genes of miR‐148b in muscle cells and were both downregulated during the transition toward inactive status.

### Muscle cell modulation of miR‐148b affected insulin‐signaling pathway and glucose uptake

**Figure 4:**
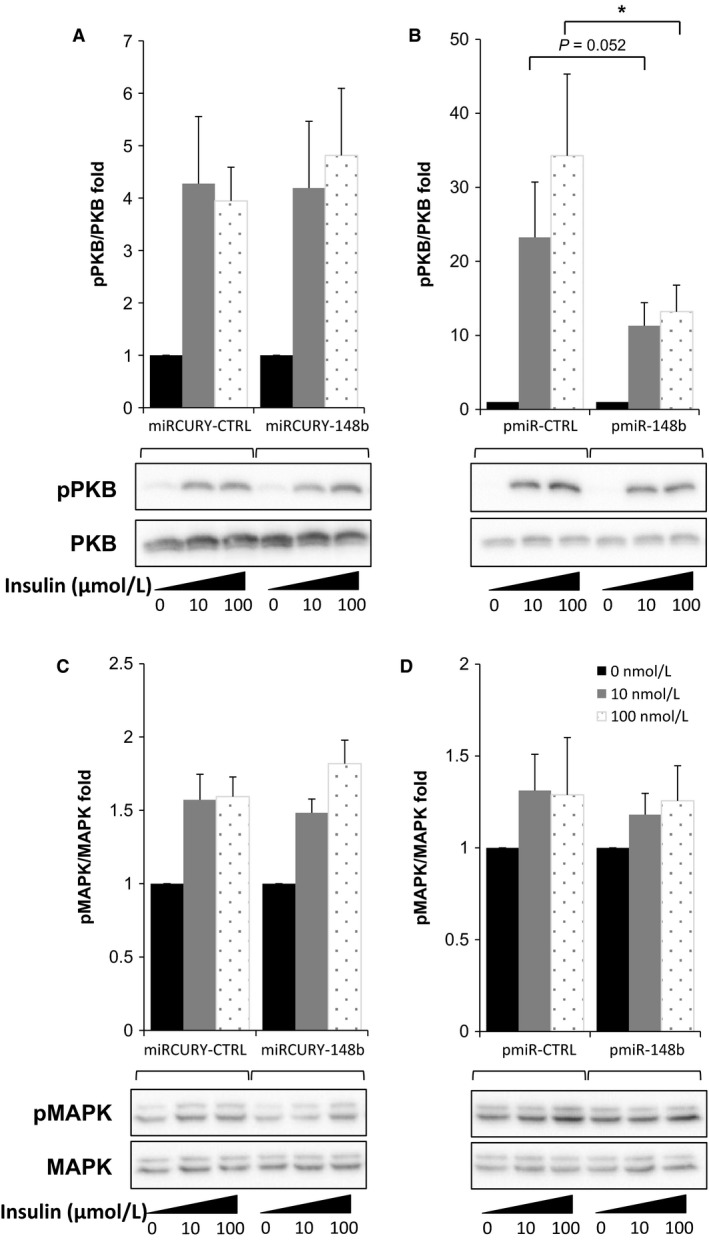
Increase in miR‐148b in muscle cell alters insulin signaling. Human primary myotubes were transfected with anti‐miR‐148b (miRCURY‐148b) or anti‐miRcontrol (miRCURY‐CTRL), and with a plasmid expressing pre‐miR148b (pmiR‐148b) or a control plasmid (pmiR‐CTRL). Phosphorylation of PKB and MAPK in response to 10 nmol/L and 100 nmol/L insulin was quantified by Western blotting. Results are presented as fold changes in pPKB/PKB ratios after miR‐148b inhibition (A) or overexpression (B), and as pMAPK/MAPK ratios after miR‐148b inhibition (C) or overexpression (D). Values are means ± SEM for *n* = 6 experiments. *indicates *P* < 0.05 compared to control conditions.

We next studied the effects of miR‐148b modulations on insulin response assessed by quantifying the level of phosphorylation of PKB and MAPK proteins in response to insulin treatment. Compared to control conditions, inhibition of miR‐148b in human myotubes had no effect on PKB phosphorylation in response to 10 nmol/L and 100 nmol/L insulin (Fig. 4A), whereas overexpression of miR‐148b decreased PKB phosphorylation (−52%, *P *=* *0.05 and −62%, *P *<* *0.05, respectively) in response to insulin (Fig. 4B). When examining the MAPK pathway, modulation of miR‐148b content in human myotubes did not modify p42/44 phosphorylation status, whether miR‐148b was inhibited or increased (Fig. 4C and D).

**Figure 5:**
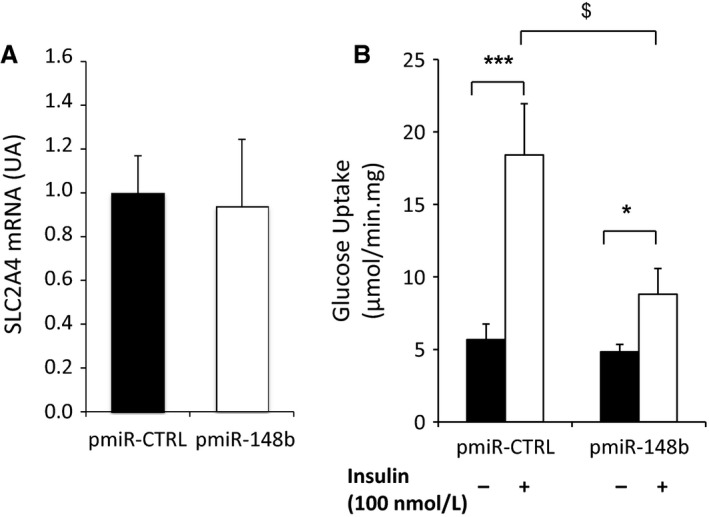
Overexpression of miR‐148b inhibits glucose uptake in muscle cells. Human primary myotubes were transfected with a plasmid expressing pre‐miR‐148b (pmiR‐148b) or a control plasmid (pmiR‐CTRL). (A) mRNA quantification of SLC2A4 (Glut4) gene after control and miR‐148b overexpression in human myotubes. Values are means ± SEM for *n* = 3 different experiments with internal replicates; (B) Glucose uptake was quantified after 1 h insulin stimulation. Values are means ± SEM for *n* = 8 replicates. *indicates *P* < 0.05; ***P < 0.001 when comparing insulin versus noninsulin stimulated cells; and ^$^ *P* < 0.005 when comparing insulin fold increase in pre‐miR‐148b versus control transfected myotubes.

To demonstrate that the increase in muscle miR‐148b content metabolically affects the cell response to insulin, we finally measured the amount of glucose uptake upon insulin stimulation. As shown in Figure 5, although no change in Glut4 expression is observed (Fig. 5A), overexpression of miR‐148b in human myotubes dramatically decreased glucose uptake in response to insulin (Fig. 5B). Compared to the increase in glucose uptake upon insulin stimulation in control cells, miR‐148b‐overexpressing cells showed a significantly reduced fold change in glucose uptake in response to insulin (1.8‐fold vs. 3.3‐fold), confirming that accumulation of miR‐148b impaired insulin responsiveness.

## Discussion

Physical inactivity and sedentary lifestyle affect the whole body and lead to the development of metabolic chronic disease, in particular diabetes [1–4]. The molecular alterations occurring in the skeletal muscle tissue during the early phase of transition from active to inactive status are, however, not completely understood. The characterization of key events involved in the development of insulin resistance in muscle is of major relevance, given the role of this tissue in whole‐body glucose homeostasis. Thus, in this study, we have analyzed for the first time the role of miR‐148b in skeletal muscle during the early phase of transition toward physical inactivity in human volunteers and mice.

We showed that decreasing physical activity lead to an increase in miR‐148b muscle content in human and mice, whereas the opposite transition (toward activity) did not affected muscle miR‐148b content. The specific protocol conditions (mild intensity of the changes may explain the lack of change for miR‐133a that had been observed in more severe conditions as bed rest (Ringholm et al. 2011a) or acute exercise (Russell et al. 2013)). Rodent wheel‐lock protocols have been used to demonstrate insulin sensitivity reduction at both muscle (Kump and Booth 2005) and whole body levels (Teich et al. 2016). Our data in mice are in agreement with previous studies showing that muscle disuse also lead to an increase in miR‐148b level in rats *soleus* muscle after 7 days of hind‐limb suspension (McCarthy et al. 2009). However, miR‐148b muscle content was found slightly decreased in a 10‐day bed rest in human (Rezen et al. 2014). This discrepancy could be related to differences in time course, duration, and/or intensity of the protocol, the bed rest inducing more severe metabolic changes than the induction of an inactive behavior.

In order to determine whether miR‐148b might affect muscle cell metabolism, we modulated its level in human primary myotubes and analyzed the regulations of two known target genes (i.e., NRAS and ROCK1). Our data showed that overexpression of miR‐148b decreased NRAS and ROCK1 protein levels in muscle, whereas inhibition of miR‐148b induced opposite effects. These two proteins had already been identified as miR‐148b targets in aggressive breast tumor (Cimino et al. 2013) and, for ROCK1, in hepatocellular carcinoma cells (Chen et al. 2016).

We further found that the decrease in NRAS and ROCK1 proteins, induced by overexpression of miR‐148b, was associated with PKB phosphorylation alteration and reduced glucose uptake in response to insulin, in human muscle cells. This is in line with previous studies demonstrating that ROCK1 deficiency causes insulin resistance by impairing insulin signaling in skeletal muscle in mice (Lee et al. 2009) and plays an important role as positive regulator of insulin action on glucose transport in adipocytes and muscle cells (Chun et al. 2012) or of PKB activity (Peng et al. 2015). It was shown that miR‐148b is involved in the PKB pathway by downregulation of ErbB3 receptor (Bischoff et al. 2015). These results have further relevance, as Chun et al. (2011) shown a disruption of ROCK1 activity in response to insulin in muscle of type 2 diabetes obese subjects, highlighting the role of ROCK1 in the development of insulin resistance in muscle. The ras proteins are downstream actors of the insulin‐signaling pathway, activated by the Grb/SOS complexes. In turn, they activate the kinases Raf, MEK, and MAPK (see Siddle 2011 for review). Inhibition of Ras is a target in cancer research, aiming at limiting cell growth and proliferation (for review see Baines et al. 2011). How NRAS downregulated impacted the insulin‐signaling pathway remain to be characterized, as it has also be shown that Ras inhibition could induce insulin sensitivity and glucose uptake in a C2C12 mouse muscle cell model (Mor et al. 2011). Meanwhile, the MAPK signaling pathway was not affected by miR‐148b suggesting that the downregulation of NRAS protein had fewer impacts on downstream effect like cell growth or differentiation in muscle cells, despite the validation of NRAS as a target gene in the muscle cells.

Interestingly, we found that only the transition toward inactive status, but not the transition from inactive to active status, modified skeletal muscle miR‐148b levels. We also showed that only overexpression and not downregulation of miR‐148b affected the insulin‐signaling pathway in muscle cells. This suggests that the molecular events in skeletal muscle involved in response to transitions toward physical inactivity are distinct from those involved in response physical activity. In line with these results, we found that the deleterious effect of detraining on dietary fat oxidation and trafficking in men was more marked than the beneficial effect of training (Bergouignan et al. 2013). Taken together these results support the hypothesis of Hamilton et al. of the existence of an inactivity physiology that is distinctive from an exercise physiology (Hamilton et al. 2007).

**Figure 6:**
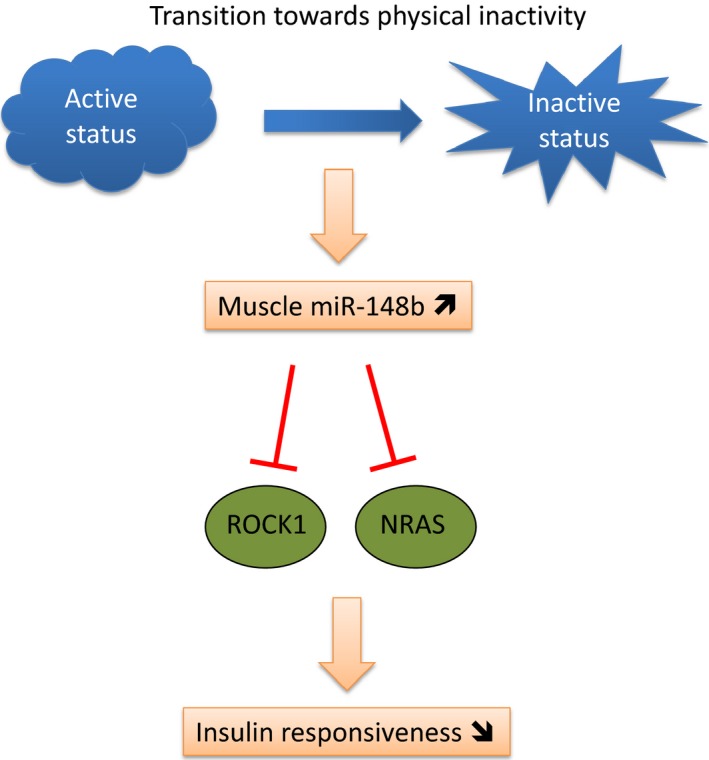
Diagram representing the effects of the early transition toward inactivity on skeletal muscle cell miR‐148b content and their consequences on insulin‐signaling pathways and glucose uptake that may affect whole‐body insulin sensitivity and glucose homeostasis.

In conclusion, our data support the global hypothesis that the early phase of transition toward inactivity leads to acute changes in muscle metabolism which are different from those involved in physical activity. We further highlighted the specific role of muscle miR‐148b in these muscle changes (Fig. 6) that could participate in the whole‐body insulin sensitivity degradation. The molecular actors involved in the transcriptional regulation of miR‐148b in muscle have now to be identified in order to firmly confirm its role in glucose homeostasis in relation to physical inactivity.

## Conflict of Interest

None declared.

## Supporting information




**Figure S1.** Mouse body weight for the SED, EX WL2 and WL4 groups during the protocol. Wheels were locked for WL2 and WL4 after 8 weeks of free access. WL2 mice were sacrificed at age of 20 weeks.
**Figure S2.** Mean running distance for the SED, EX WL2 and WL4 groups during the protocol. Wheels were locked for WL2 and WL4 after 8 weeks of free access.
**Figure S3.** Food intake for the SED, EX WL2 and WL4 groups during the protocol. Wheels were locked for WL2 and WL4 after 8 weeks of free access. WL2 and WL4 mice spontaneously reduced their food intake after wheel lock.
**Figure S4.** miR‐133a quantification by RT‐PCR in the LIPOX protocol. Measurements were in the same conditions and for the same samples as described for miR‐148b in figure 2A. For miR‐133a, no significant changes were observed between groups.
