# Supplementary material for: Transition from physical activity to inactivity increases skeletal muscle miR‐148b content and triggers insulin resistance
Source: Physiol Rep. 2016 Sep 5;4(17):e12902. doi: 10.14814/phy2.12902 (PMC5027343; doi:10.14814/phy2.12902)
Supplement: Supplementary file 1 — Figure S1. Mouse body weight for the SED, EX WL2 and WL4 groups during the protocol. Wheels were locked for WL2 and WL4 after 8 weeks of free access. WL2 mice were sacrificed at age of 20 weeks. Figure S2. Mean running distance for the SED, EX WL2 and WL4 groups during the protocol. Wheels were locked for WL2 and WL4 after 8 weeks of free access. Figure S3. Food intake for the SED, EX WL2 and WL4 groups during the protocol. Wheels were locked for WL2 and WL4 after 8 weeks of free access. WL2 and WL4 mice spontaneously reduced their food intake after wheel lock. Figure S4. miR‐133a quantification by RT‐PCR in the LIPOX protocol. Measurements were in the same conditions and for the same samples as described for miR‐148b in figure 2A. For miR‐133a, no significant changes were observed between groups. [file PHY2-4-12902-s001.pptx]

## Slide 1
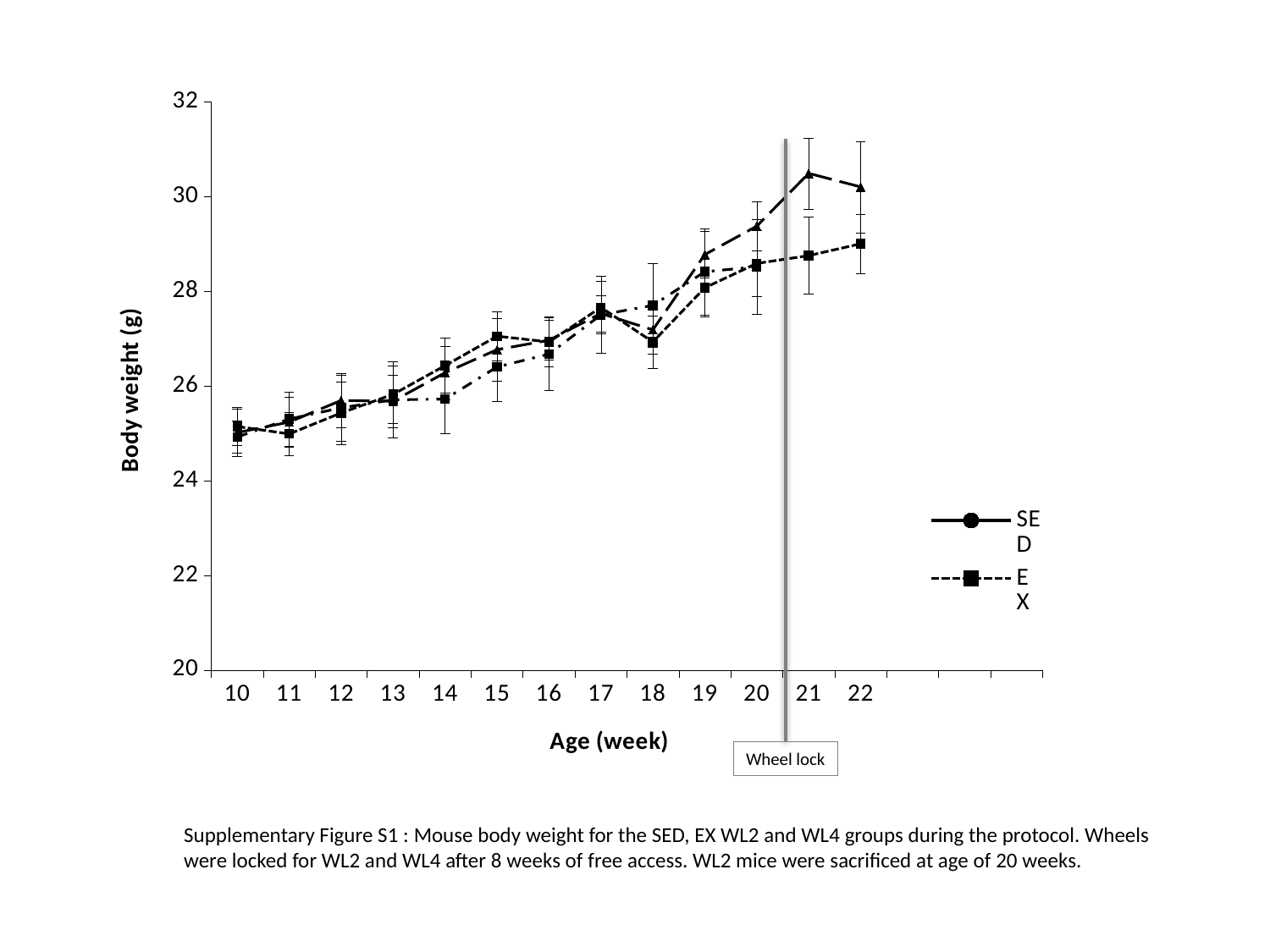

[unsupported chart]
Wheel lock
Supplementary Figure S1 : Mouse body weight for the SED, EX WL2 and WL4 groups during the protocol. Wheels were locked for WL2 and WL4 after 8 weeks of free access. WL2 mice were sacrificed at age of 20 weeks.

## Slide 2
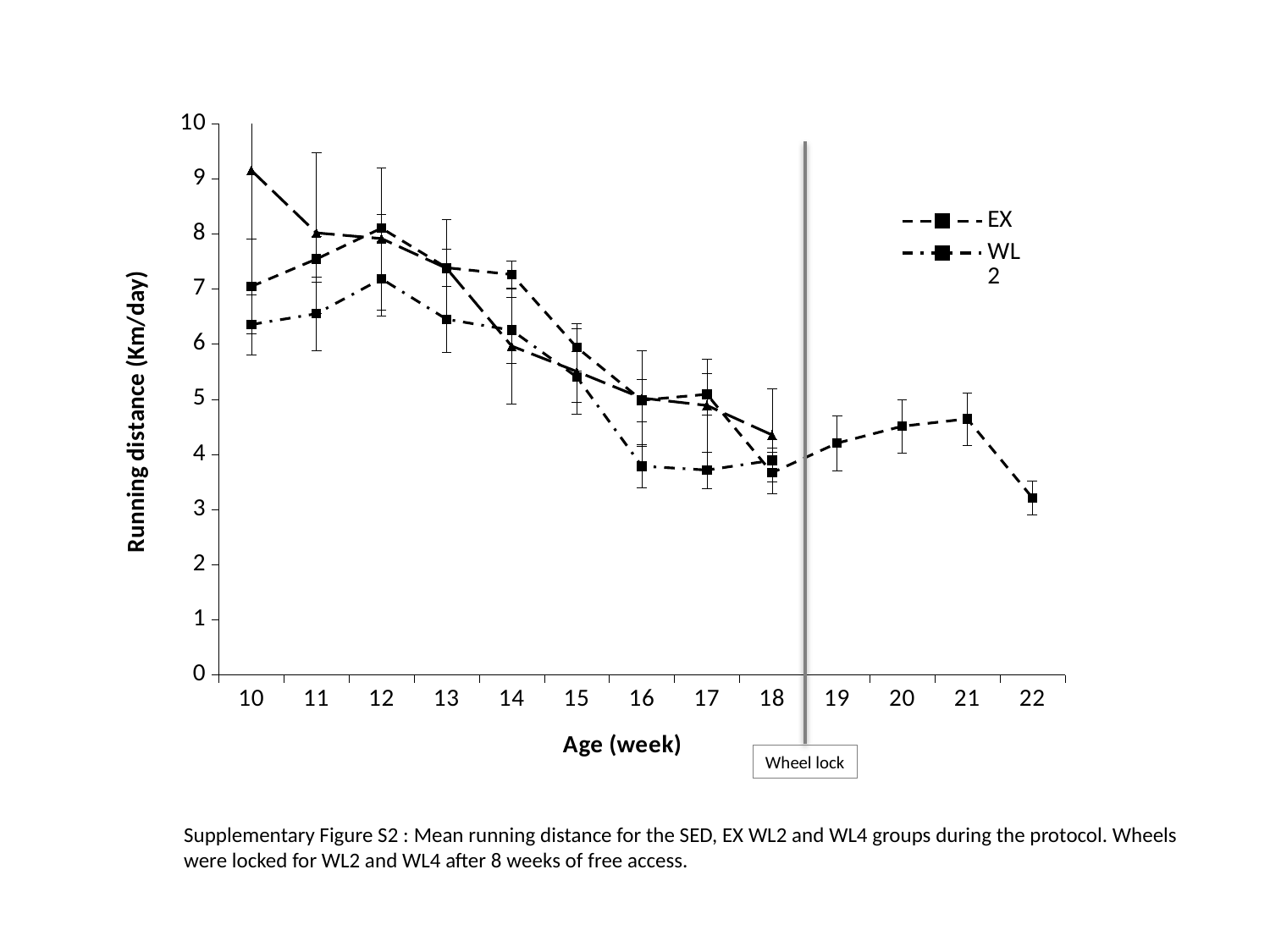

[unsupported chart]
Wheel lock
Supplementary Figure S2 : Mean running distance for the SED, EX WL2 and WL4 groups during the protocol. Wheels were locked for WL2 and WL4 after 8 weeks of free access.

## Slide 3
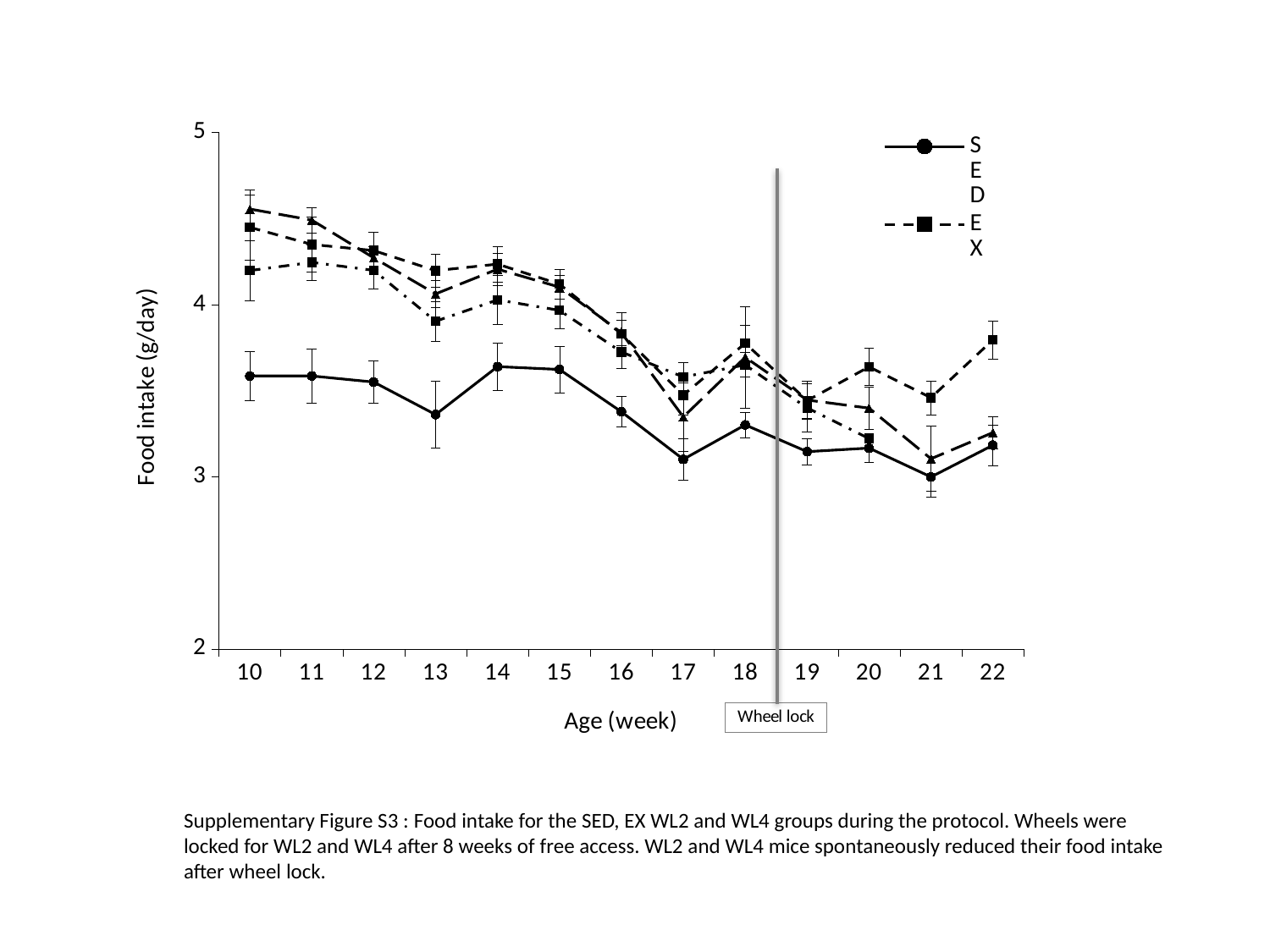

[unsupported chart]
Supplementary Figure S3 : Food intake for the SED, EX WL2 and WL4 groups during the protocol. Wheels were locked for WL2 and WL4 after 8 weeks of free access. WL2 and WL4 mice spontaneously reduced their food intake after wheel lock.

## Slide 4
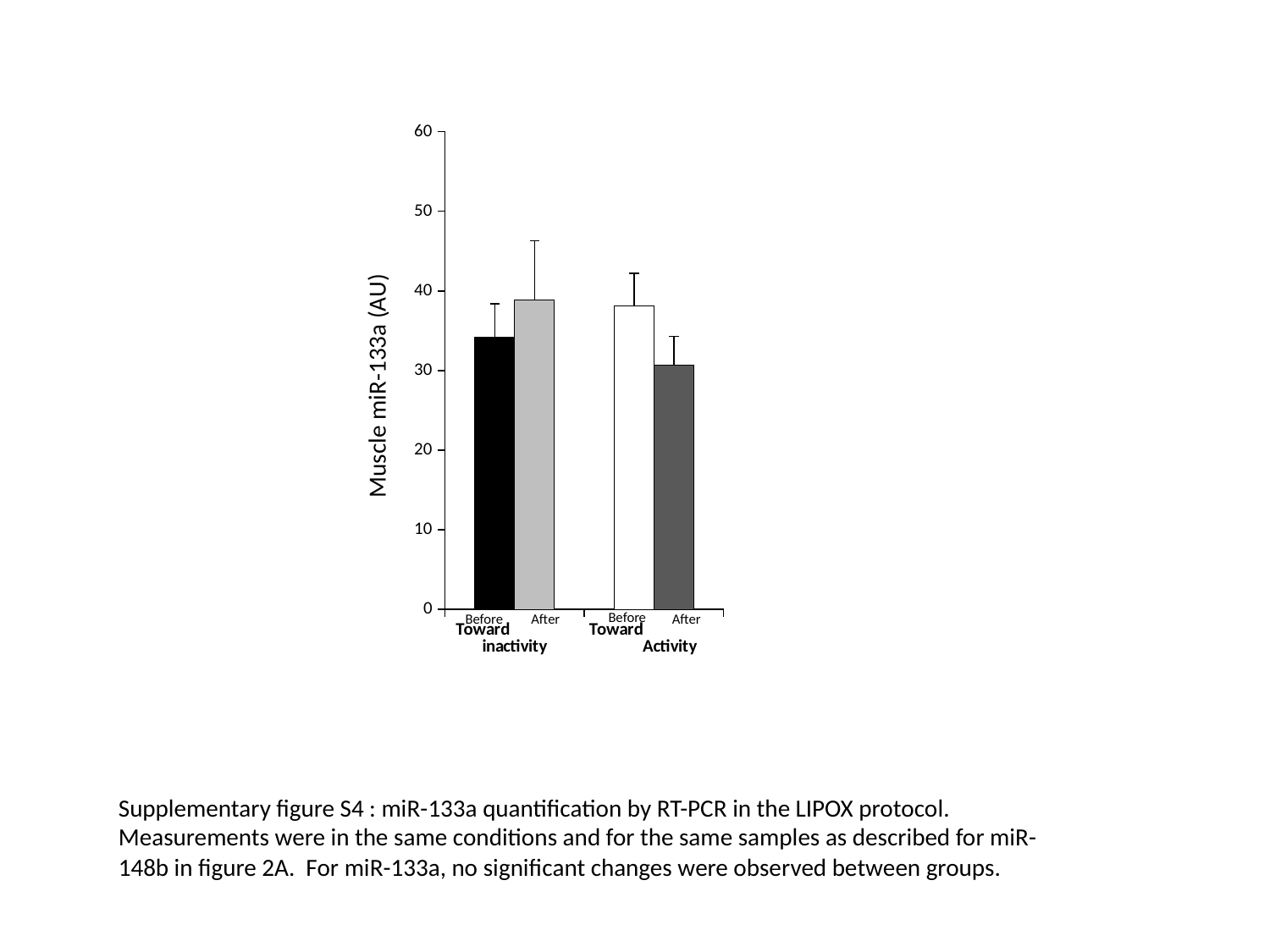

### Chart
| Category | Before | After |
|---|---|---|
| Toward inactivity | 34.1842157122222 | 38.80729584571429 |
| Toward Activity | 38.07314679571429 | 30.6605932171429 |Muscle miR-133a (AU)
Before
Before
After
After
Supplementary figure S4 : miR-133a quantification by RT-PCR in the LIPOX protocol. Measurements were in the same conditions and for the same samples as described for miR-148b in figure 2A. For miR-133a, no significant changes were observed between groups.
